# Dr. Murphy and the “Christian Hospital” Schemers

**Published:** 1903-07

**Authors:** 


### DR. MURPHY AND THE "CHRISTIAN HOSPITAL" SCHEMERS.

I was much chagrined to learn, when it was too late to correct or suppress it, that in the June number of this JOURNAL, under the heading, "A Sheep-shearing Trust," I unintentionally and unwittingly did Dr. Murphy a great injustice. Murphy had nothing to do with the villainous scheme, but was victimized by a lot of sharpers—one of whom, at least, it is stated in the *Chicago Record Herald* (June 5th), has served a term in the penitentiary for swindling. The *Record Herald* publishes a cut of the fellow with his prison number 6843. It is said that these fellows are the gang that promulgated a similar scheme at Niles, Michigan, for fleecing the afflicted and hoodwinking the vain and credulous doctors throughout the country, and that they were run out of there by the law. I never heard of any of them before, by name, and just assumed that they were a lot of Chicago unknown sharpers, who had tempted Murphy with a plausible and dazzling scheme to "get rich quick," and seduced him; that it was another case of "a good man gone astray." The very boldness and audacity of the manner in which they used Murphy's name—a copy of his autograph being affixed to the "certificates"—misled me, and I neglected to write to him, as I see now that I should have done, before proclaiming him "President." I thought it was unnecessary; it looked like too clear a case; it looked conclusive. *Prima facie* the evidence was against him. I learn from a letter from the President of the Chicago Medical Society that that society is doing what they can to convict those fellows, especially Probert and Wood—that is, they are helping the U. S. postoffice authorities to do so; that Probert and Wood are to be tried in October. The writer of the letter (Dr. W. A. Evans, President of the society and chairman of its Medico-Legal Committee) says: "They (P. and W.) are now out under bond. Will you help us to gather evidence in Texas, and will you come to the trial in October? * * * Will you help us to make the Texas doctors stick, for now they are buying off witnesses by paying back their money."

I tender Dr. Murphy my sincere apologies. I had neglected to read my exchanges that month (for reasons that readers will understand) and was not aware of the facts in the case. I was influenced to write as I did by the reasons stated above and by an editorial in the *New York Medical Record* (June 16th), in which the writer assumed, as I did, that there was no doubt of the connection with the concern, as president, of a "certain very distinguished surgeon." I will be glad to undo the wrong I have thus done Murphy, and will gladly co-operate with the profession in Chicago in getting up evidence. I wish, therefore, that all readers of this who have received the circulars and decoy "Christian Red Cross Hospital Certificates" and invitations to walk in and be skinned would send them to me. I will forward them to the Chicago Medical Society. The following letter from Dr. G. F. Lydston is self-explanatory, and is here published at his request:

"CHICAGO, ILL., July 6, 1903.

"*Dr. F. E. Daniel.*

"DEAR SIR: I note in your 'Red Back' an editorial flaying 'The Christian Hospital' fake, and incidentally Dr. John B. Murphy. I am astounded that you should convict Dr. Murphy of complicity in that nefarious scheme. He not only had no part in the infernal fake, but has been instrumental in bringing to justice the scoundrels who surreptitiously used his name. Why, my dear Daniel, you surely don't think that Murphy is an idiot. No man of intelligence would countenance a scheme that would be simply professional suicide. Murphy has been the victim of a lot of ex-jail birds and others who should be in jail now. Believe me, my dear Daniel, you have fired your cannon prematurely and hit a man who was already hurt to the quick by the malodorous conduct of a lot of rascals. If I thought Murphy capable of such an idiotic, scoundrelly piece of quackery, I'd scratch him off my list. As it is, I am proud to consider him my friend and to be his friend.

"Will you not publish this and thus make reparation?

"Your friend,

"G. Frank Lydston."

